# Evaluation of a manualised speech and language therapy programme for children with social communication disorder: the SCIP feasibility study

**DOI:** 10.1186/s40814-020-00658-2

**Published:** 2020-09-22

**Authors:** Catherine Adams, Jacqueline Gaile, Hazel Roddam, Janet Baxendale, Laura Clitheroe, Richard Emsley

**Affiliations:** 1 Division of Human Communication, Development and Hearing, School of Health Sciences, Ellen Wilkinson Building, University of Manchester, Oxford Road, Manchester, M13 9PL UK; 2 School of Health Sciences, University of Central Lancashire, Preston, Lancashire PR1 2HE UK; 3 Institute of Psychiatry, Kings College, London, UK

**Keywords:** Pragmatics, Social communication, Intervention, Trial, Speech, Language therapy

## Abstract

**Background:**

Children with Social (Pragmatic) Communication Disorder (SPCD) have long-term needs in using and processing social language and have a high risk of later mental health difficulties. A manualised speech and language therapy programme, the Social Communication Intervention Programme (SCIP) provides therapy content for SPCD. A feasibility study is required to derive more precise estimates of key parameters for a future trial of SCIP.

**Aims:**

To assess the feasibility of conducting a substantive randomized controlled trial of SCIP for children with SPCD.

**Methods:**

A questionnaire was distributed to paediatric speech and language therapists in England. Survey questions addressed number of eligible children, routine intervention provision and trial recruitment factors. In the second phase, a single-arm intervention feasibility study was completed. Fifteen speech and language practitioners identified 24 children aged 5–11 years with SPCD. Practitioners received training/supervision to deliver 20 SCIP therapy sessions to each child. At time 1, parents of participating children provided three communication goals; expected steps in each goal were defined. After intervention, parents and practitioners independently rated each goal compared to baseline ability. Two research practitioners compared parent post-intervention commentaries with outcome scores to derive guidance about clinical significance. All practitioners recorded audio commentaries on therapy experiences. Post-intervention interviews were conducted with 6 practitioners and 6 parents. An expert panel completed a Delphi consultation on trial design.

**Results:**

Routine practice for SPCD varies widely. Children tend to be embedded in autism provision. Participation in a future trial was well supported provided resources are available to services. Outcomes analysis indicated all children except one made some progress on parent ratings; all children made progress on practitioner ratings. A power analysis for a future trial was carried out using current outcome measure as putative primary endpoint. Practitioners’ audio-diaries provided suggestions for training and adaption in a future trial. Outcomes and therapy methods were acceptable to practitioners and parents.

**Conclusions:**

The feasibility study evaluated a novel outcome measure of social communication skills in SPCD. A power calculation indicated a feasible framework for a trial within a realistic period of time. Recommendations for recruitment methods, adaptation of manual and training were supported by practitioners and an expert panel.

**Trial registration:**

Title: Speech-language therapy for child social communication disorder

Trial ID: ISRCTN48030419. Date registered: January 1, 2017. Registered retrospectively.

## Background

The presence of significant and persistent social communication difficulties in middle childhood is associated with adverse outcomes such as behavioural difficulties in adolescence [1], in sustaining peer relations [2], successful employment [3] and with later mental health conditions [4]. This type of communication impairment comprises disproportionate difficulty with pragmatics (the social use of language) [5] and some structural language impairments [6, 7]. Speech and language therapists (SLT) therefore have a key role in identifying and managing the social communication needs of these children as a contribution to prevention of negative outcomes. However, despite a call for more research on pragmatic language intervention in the relevant systematic review [8], there are no clinical trials available [9].

In order to progress to a better state of evidence, it is necessary to gauge feasibility and identify real and potential barriers to a substantive trial. Known potential issues are the identification of children with social communication difficulties, consensus on what treatment as usual (TAU) is, whether a novel complex social communication intervention is acceptable and can be implemented by SLT practitioners in schools and clinics, and how changes in social communication and pragmatics can be measured.

The literature describes two groups of children who have significant and persistent social communication difficulties with a specific focus on pragmatic impairment. Children with high-functioning autism spectrum disorder (HFASD) have heterogeneous pragmatic deficits [10] and long-term language processing difficulties [11]. A related group of children, termed Social (Pragmatic) Communication Disorder (SPCD), have similar pragmatic and language impairments [12] but may lie just below the threshold for ASD diagnosis [13]. It is possible that both these groups might benefit from social communication therapy but at present, we do not know precisely what routine SLT therapy services are provided for either of them. This knowledge is required to construct an alternative treatment condition to any novel programme in a clinical trial.

Practitioner guidance on intervention approaches for children with SPCD is provided by professional bodies, and there is a substantial descriptive therapy literature [14]. The American Speech and Hearing Association (ASHA) lists a number of intervention programmes appropriate for school-age children with SPCD, which fall under the broad heading of social skills interventions. Gerber et al. [9] examined the evidence regarding conversation/pragmatics intervention for children who have SPCD and found small-scale studies only, with variation in content and goals of treatment, reflecting the diverse nature of communication needs within the group. Gerber et al.’s review lamented the absence of theoretical underpinning of intervention methods and the difficulties of generating clinical guidance in the context of limited evidence.

In our previous work, we have developed a theoretically driven, manualised intervention, the Social Communication Intervention Programme (SCIP), [15] specifically for children with social communication difficulties. The manual includes a method for individualisation of therapy for heterogeneous pragmatic and language needs as well as therapy activities/resources. In a small-scale school-based trial [16], Adams and colleagues found an advantage of SCIP intervention over routine treatment on outcomes shown to be of value to parents/carers: carer-rated pragmatic competence and changes in social communication and language skills, teacher-rated learning skills and an observational measure of conversation skills [17]. However, no changes in language functioning using standardised language tests were shown.

A traditional approach to outcome measures using impairment measures may therefore not capture changes in communication which are of importance to service users for this group of children [18]. In addition, evaluating outcomes of pragmatic interventions has proved to be problematic due to the lack of valid and reliable measures of pragmatics and conversational skills [19]. Given language/pragmatics heterogeneity at baseline, a way forward in a clinical trial of social communication intervention may be to adopt an individualised approach to therapy planning and a preference-based outcome measure. We propose to evaluate the feasibility of using a modified goal attainment scaling (GAS) [20] as a primary endpoint in a clinical trial of SCIP. There is evidence in favour of the use of GAS, over standardised measures, for sensitivity to clinically significant change [21] but it has not previously been explored as an outcome in social communication intervention studies. In addition, we will address the notion of how clinical significance, as observed by service users, relates to such a scale. Kazdin defines clinical significance as “the practical or applied value or importance of the effect of an intervention” [22]. In the current study, we were interested in which observed changes in communication behaviour coincided with service users’ views on progress. This may be an important factor in implementing GAS in a larger project.

The drive for evidence-based preventative actions, service user feedback and preliminary findings imply that a full-scale clinical trial of SCIP may be indicated. A feasibility study is required to derive more precise estimates of the proposed primary endpoint, level of adherence to the SCIP treatment protocol, service providers’ and practitioners’ views on acceptable models of delivery, as well as estimates of recruitment, retention and response rates.

## Aims and objectives

To assess the viability of conducting a substantive randomized controlled trial of SCIP for children aged 5–11 years who have significant social communication needs, to survey current service SLT provision and intervention methods used in England for these children, to refine a novel intervention for delivery in routine clinical practice, to estimate parameters for a randomised controlled trial of the new social communication intervention, to estimate sample size by studying variability of a modified goal attainment scaling (GAS) as primary endpoint, to explore training and acceptability of the intervention and to obtain expert consensus on key parameters for a trial.

## Methods

The study was carried out in three phases in sequential order.

### Phase 1

#### Aims

To acquire information on the nature of current routine SLT practice for children with social communication needs, to identify views on training and support needs of practitioners to implement SCIP in a clinical trial, to explore practitioner willingness to participate/be randomized in a trial, to estimate the number of eligible child participants for a clinical trial and to obtain opinions on key recruitment and participant factors for a substantive trial.

#### Method

An online open invitation questionnaire was distributed to UK National Health Service (NHS) SLTs, and NHS SLT service leads in England and two independent SLT practitioners working in private clinics or local authority maintained schools (non-NHS). 103 SLTs consented to participate. Of these, 76 complete survey responses were obtained (51 NHS only, 23 non-NHS only, 2 both NHS and non-NHS). The sample contained eight NHS and three non-NHS service managers, all of whom held a clinical caseload. Response rates were similar to other e-surveys of specialist SLT. Survey questions addressed views on issues identified in the phase 1 “Aims” section above.

### Phase 2

#### Aims

To estimate the recruitment/retention rates needed to collect completed data in a main trial, to estimate response to questionnaire rates needed to collect completed data in a main trial, to estimate rates of practitioner adherence to the intervention, to refine the characteristics of a modified goal attainment scale (SCIP-GAS) primary outcome measure for effective use with the target population and to estimate variability of the primary endpoint to inform sample size calculations for a substantive randomised trial.

### Phase 2 method

This was a small-scale, single-arm feasibility study. Practitioner recruitment: SLT practitioners, who routinely treat children with SPCD, were recruited via the research team’s established links across the northwest of England, the NIHR Greater Manchester Clinical Research Network and the research team’s national network. All practitioners worked within the North West of England, except two independent practitioners from the south of England. Practitioners were required to have at least 2 years’ experience of intervention for children with communication disorders and to be willing to participate in SCIP training and intervention delivery. Each practitioner contacted at least one family of an eligible child in order to recruit and gain consent for participation. All practitioners were experienced SLTs except one, who was a special needs teacher with a specialism in language support. A sample of 15 practitioners and children was considered sufficient to assess the feasibility criteria and is large enough to estimate the variance of the primary outcome measure to inform a sample size calculation for a substantive trial design [23]. Practitioner and child/family participants were recruited into the study between September 2016 and October 2017. Baseline assessments took place during this period. The final follow-up assessment took place in April 2018.

Child participant inclusion criteria were as follows: aged between 5 years and 0 months and 10 years 11 months; parents/carers able to participate in minimum of five intervention sessions; non-verbal performance on Ravens Coloured Progressive Matrices [24] centile ≥ 5; score in the communication impaired range (< 55) on the Children’s Communication Checklist-2 General Communication Composite (CCC-2) [25]—a parent report measure of language and social communication skills; social communication problems as observed by the practitioner, defined as a minimum of two out of five social communication difficulties on the SCIP social communication checklist (SCIP-SCCheck), based on characteristics listed in previous clinical descriptive accounts [26] (see Appendix 1 for checklist). Exclusion criteria were as follows: severe speech unintelligibility/deafness, severe conduct/hyperactivity disorder which precludes engagement with the intervention and cases where child has no knowledge of English as a spoken language.

For practitioner training, in order to refine SCIP for practitioner implementation, a programme of training for practitioners was devised and implemented by a research speech and language therapist (RSLT) who was also responsible for all intervention supervision. Training content was delivered via a 1-day workshop and comprised pre-course reading on the theoretical rationale, the overall structure and principles of SCIP delivery; rationale for assessing language, social cognition and pragmatics; planning therapy using Assessment-to-Intervention Mapping method in manual; setting goals from parent priorities and involving others in therapy delivery.

For phase 2 intervention, practitioners received a copy of the manual, some therapy resources and 6 h of supervision from the RSLT across the intervention period. Initial goals of intervention were refined jointly with the RSLT. Practitioners delivered intervention with the child in school or at home up to a maximum of 20 direct therapy sessions. Therapy commenced within 1 month after baseline assessment. Liaison with school and family was conducted at the practitioner’s discretion and availability of others, using written means or meetings to share information.

For baseline and outcome measures, a researcher independent of the intervention completed other language assessments for the purpose of intervention planning: Clinical Evaluation of Language Fundamentals (CELF-4) [27] and Assessment of Comprehension and Expression (ACE) [28]. The ADOS-2 Module 3 [29] was completed for indicative assessment of ASD. The primary endpoint was the SCIP Goal Attainment Scale (SCIP-GAS). Parents provided three priority areas for intervention at baseline assessment (time 1). In discussion with the RSLT and practitioner, three goals for the SCIP intervention period were set to reflect these priorities. The SCIP-GAS form set out the parent priority, the baseline level of ability and the goal (desired ability) after an intervention (see Appendix 2 for a sample SCIP-GAS form). After intervention (time 2), parents used the SCIP-GAS form to rate their child’s progress. After defining GAS goals at mapping and at outcome (T2), the parent rated each goal compared to T1 as follows: − 1 = got worse, 0 = no change, + 1/+ 2 = partial achievement, + 3 = fully achieved, + 4 = slightly exceeded and + 5 greatly exceeded. Practitioners completed a SCIP-GAS outcome form at Time 2 for each child independently of the parent.

The analysis consisted of simple descriptive statistics presented as means (SD) for continuous variables or count and percent for categorical variables.

For refinement of SCIP-GAS procedure, our exploration of SCIP-GAS as a potential endpoint included an analysis of what scores would constitute clinical significance. In order to do this, two of the investigators (both senior research SLTs) examined the range of SCIP-GAS numeric outcomes and linked these with parent narrative comments from the post intervention SCIP-GAS form to derive guidance about clinical significance. This was important to allow for confirmation of which GAS values were associated with notable functional change.

For adherence to intervention manual, adherence during intervention was by (a) RSLT’s analysis of therapy sessions of practice against the model therapy activity and (b) analysis of practitioners’ reflective audio-diary of what content was delivered, how the delivered content adhered to the manualised version and a short commentary on any difficulties or successes in delivering the intervention in routine practice. Planned versus delivered was completed on 30% of the sample of child participants, and observation was completed with 5 practitioners (33%).

### Phase 3

#### Aims

To explore factors associated with training and acceptability of the intervention to all stakeholders and to obtain consensus on key parameters for a full trial.

#### Method

For reflections of participating SLTs, practitioners were provided with an audio recording device at the start of intervention. They were asked to make short oral notes regarding the content and progress in each therapy session. Additional notes about changes to plans, changes or adaptations to the intervention procedure or therapy activity or regarding the child’s response to intervention were requested. Practitioners’ diary entries comprised a combination of audio recordings plus written contributions. These were analysed using a Framework Analysis [30] by one member of the research team. Codes were defined and recorded incrementally for each participant, which allowed comparison of the descriptive content themes across all participants.

For interviews with practitioner and parents, interviews were conducted either at the mid-point or immediately after intervention with six SLTs and six parents of children involved in the study, to ask about their experience of participating in the study and of SCIP intervention. A topic guide was developed and used in all interviews. Practitioner interviews topic guide covered SCIP training, supervision, GAS goal setting process, overall content and purpose of therapy; putting SCIP therapy into practice. Parent interviews topic guide covered expectations from the intervention, experience of setting goals for therapy, experience of therapy and any changes noticed in the child, the family and/or at school.

For Delphi consensus procedure, towards the end of the study, a two-round Delphi method consultation was conducted in which an expert panel of SLT practitioners and managers were surveyed for their views on a series of statements relating to potential design and implementation of a clinical trial. For each statement, a paragraph explaining the rationale for the statement, based on information that had been compiled from research activities and/or theoretical support was provided. Round 1 responses and comments were analysed, and statements amended where necessary (where consensus was not reached) and resubmitted to the panel in round 2. Consensus was defined as 80% of respondents selecting either ‘Partially Agree’ or ‘Strongly Agree’.

## Results

### Recruitment and retention for future trial

Routine practice for this population (the potential TAU condition in a future trial) was found to vary widely in our survey of SLT practitioners in England. The majority of practitioners (75%) delegate therapy delivery to school teaching assistants; 30% of therapy across NHS and non-NHS provision is delivered by SLT assistants. Weekly individual and group sessions of 30–60 min duration are the most common therapy delivery option. Some services did not deliver any therapy at all or provided a fixed number of sessions in an episode of care model. The number of intervention resources or approaches used in current practice was very large; 56 intervention approaches were described in a sample of 54 practitioners. A large majority of respondents to the survey would be willing to be randomised in a future trial but time available would be a major barrier to participation. Twenty-three percent of SLTs would be willing but unable to participate. Fifty-five percent of respondents agreed that a trial was an important method to show effectiveness of a new intervention. Other points relevant to recruitment are made in the Delphi study findings below.

Training and support needs for a future clinical trial were identified in the survey and later confirmed in practitioner diary analysis and the Delphi study. Forty-three percent of phase 1 survey respondents stated that they would require support and training in recruitment of child participants in a future trial. Dedicated funding and time away from routine duties was specified as a support need by 26% of respondents. Other requirements were listed as provision of information for parents and support from service leads. The majority of survey respondents would require support for involving others in intervention, permission from service lead to participate, opportunity to deliver the intervention flexibly and ability/time to share information with teachers and education support workers. Some respondents recommended being able to integrate a new intervention into an existing package of care.

**Fig. 1:**
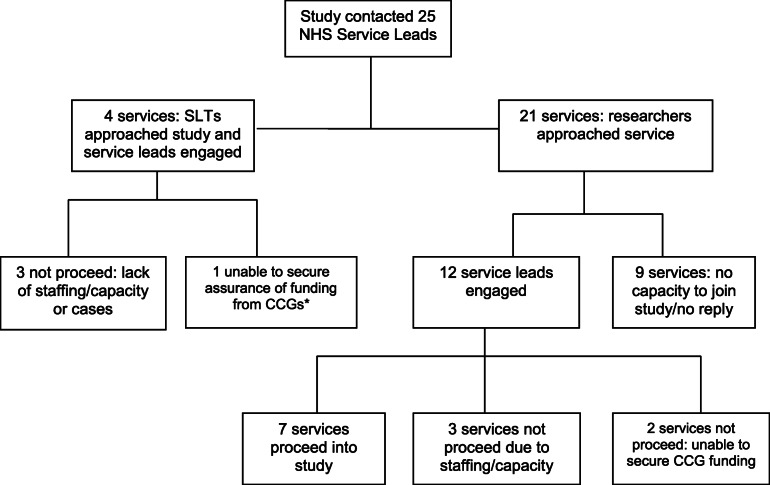
Recruitment approaches in SCIP feasibility study: NHS service level only. * CCG, Clinical Commissioning Group

In the feasibility intervention study (phase 2), SLT services and individual practitioners generally gave a positive response to recruitment requests. Of the NHS Research and Development (R&D) services approached, 50% were able to proceed into the study; R&D approval ranged from 10 days to 4 months. Reasons for not proceeding were varied but mainly based on cost and staff time. There was a highly variable approach to treatment costs across services. Recruitment progression, withdrawal and refusal reasons for NHS services only are shown in Fig. 1.

**Fig. 2:**
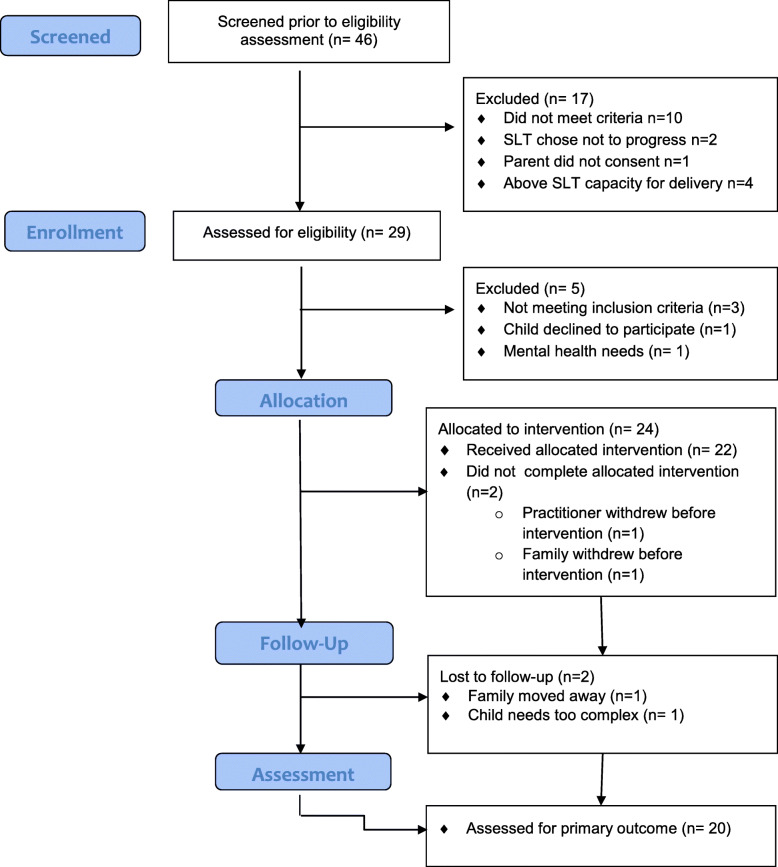
Consort diagram for SCIP feasibility intervention study

Four phase 2 practitioners were solo non-NHS SLTs; one was a specialist advisory teacher. The potential length of recruitment period per service, practitioner and child ranged from 5 weeks (non-NHS independent practitioner) to 9.5 months (NHS). Of 41 practitioners contacted, 15 practitioners were recruited, trained and started intervention; twelve completed intervention comprising 7 NHS SLT, 4 non-NHS SLTs and one specialist teacher. Dropout in NHS practitioners was 40% (illness and workload were reported as reasons); there was no dropout in non-NHS practitioners. Child participant retention and exclusions are shown in the Consort diagram in Fig. 2. Forty-six children were referred to the study; 22 children started intervention but only 20 completed. Reasons for non-progression are shown in the diagram.

Overall, the survey responses (phase 1) indicated that SPCD is not a rare condition in the population of children requiring SLT, but that these children were often included in ASD services whether diagnosed with autism or not. Therefore, SPCD may be difficult to isolate as a population. Individual responses regarding the proportion of caseload diagnosed as SPCD were too variable to be informative. An analysis of potential eligible participants was attempted from the literature and National Statistics [31]. We proceeded with caution since epidemiological studies in language disorders tend to refer to Developmental Language Disorder (DLD) or the broader Speech Language and Communication Needs (SLCN). Approaching from the SLCN angle, the population prevalence of language disorder as measured by teaching screening is 7.58% with clinically significant DLD [32]. This is equivalent to 2 children in every class of 30 pupils. However, only a proportion of these children will have SPCD. Taking 7.58% of the population of current 6 year olds in England (*n* = 729,674) provides an estimate of 55,509 children with SLCN currently. We used prevalence of a similar condition to SPCD (receptive language disorder), 4.5% [33] to conservatively estimate the proportion of children with SLCN as having SPCD. This estimates the SPCD population to be 2498 children at age six (14,987 across 6 age bands in England). Approaching from the HFASD angle where there are more robust epidemiological studies and taking 1.16% of population as diagnosed with autism [34] yields a total of 8464 with ASD, from which 32% are estimated to be high-functioning [35] which indicates 2708 children at age 6 with HFA (16,248 across 6–11 age bands).

### Outcomes of feasibility intervention study, questionnaire response rates and treatment adherence

The characteristics of children recruited into the feasibility intervention study are shown in Table 1. All children met criteria for communication impairment on the General Communication Composite of the CCC-2 and demonstrated a high number of social communication difficulties on the SCIP SCCheck. On ADOS module 3, 11 child participants met criteria for ASD; nine were defined as SPCD as they had pragmatic impairments but did not meet the autism diagnostic criteria. A wide range of scores on subtests of ACE and CELF-4 tests was observed with all mean scores in the impaired range, indicating the presence of language impairments in most children.

**Table 1:** Child participants: characteristics including language test scores at baseline

Baseline measures	*N*	Range	Mean	SD
Age (in months) at time 1	20	61–131	102	19.50
RPCM centile	20	7–95	50	32.00
CCC-2 GCC	20	21–54	33.60	9.34
CELF-4^a^ subtests				
Concepts and following directions	20	1–12	6.35	3.17
Formulated sentences	20	1–14	7.00	3.18
Word classes receptive	20	0–11	7.25	2.81
Sentence structure^b^	11	3–12	7.18	3.46
Understanding spoken paragraphs	19	2–12	7.32	3.20
ACE^a^ subtests				
Naming	20	3–12	8.10	3.02
Non-literal comprehension	20	3–14	7.05	3.15

*RPCM* Raven’s Coloured Progressive Matrices, *CCC-2 GCC* Children’s Communication Checklist-2 General Communication Composite, *CELF-4*, Clinical Evaluation of Language Fundamentals, *ACE* Assessment of Language Comprehension and Expression

^a^For the normed, standardised assessments, CELF-4 and ACE, a standard score of 10 represents the 50th centile of the population

^b^Under age 9 years only.

Parent SCIP-GAS forms were completed at home and posted back to the research team at time 2. Parents rated their children’s progress at time 2 against three goals set at baseline. Most parents completed the form independently; only one asked for a home visit to assist completion of the form. Some parents discussed the SCIP-GAS with the RSLT after they had completed it. This was largely confirmatory in nature. Practitioners rated progress compared to goals using the same scale and pre-defined steps. They completed this independently from parent and RSLT and posted their ratings directly to the researcher who was independent of the intervention.

In the intervention planning process, we stipulated that for each SCIP-GAS goal ‘expected achievement’ would score 3. Mean SCIP-GAS scores by rater are shown in Table 2. Descriptive analysis indicates all children except one made some progress on parent ratings; all children made progress on practitioner ratings. Practitioner ratings tended to be higher than parent ratings with the mean total score nearing expected achievement on all three goals. Using the sum of the achieved SCIP-GAS scores (*n* = 20), without weighing for difficulty or importance, mean parent SCIP-GAS score = 6.8 (SD 3.1) and mean SLT SCIP-GAS score = 8.6 (SD 2.2).

**Table 2:** SCIP-GAS at time 2: parent and practitioner total scores and numbers of goals that met expectation (both *n* = 20)

	Mean	SD	Range
Parent SCIP-GAS scores at Time 2	6.75	3.1	0–12
Parent: number of goals that met expectations	1.3	–	0–3
Practitioner SCIP-GAS ratings at Time 2	8.6	2.2	4–12
Practitioner: number of goals that met expectations	1.85	–	0–3

*SCIP-GAS* Social Communication Intervention Programme–Goal Attainment Scale

One of the study’s aims was to refine the GAS procedure in order to establish a meaningful outcome. Analysis of parent narrative post-intervention and the range of SCIP-GAS scores for all child participants indicated that clinical significance was associated with scores in the 6–9 point range; highly significant was associated with scores above 9 (see Table 3). These findings were used in a power calculation for a clinical trial. Further discussion and analysis of clinical significance and outcomes of pragmatics intervention are presented elsewhere [36].

**Table 3:** Analysis of association of SCIP-GAS scores with clinical significance judgements

	Not clinically significant	Borderline	Clinically significant	Highly significant
SCIP-GAS range	0–3	4–5	6–9	10–15
*N* in feasibility study	3	3	11	3

Response rates to questionnaires (parents) were uniformly high. Both SCIP-GAS and CCC-2 questionnaires were returned at 100% for time 1 and time 2 assessments. This reflects the close interaction between the research team and the parents and practitioners throughout the study.

Adherence to the intervention was high for both audit of the planned intervention content versus delivered content (adherence 92%) and adherence to therapy procedures in the SCIP manual (100%). The majority of practitioners except one (non-NHS) were compliant with supervision.

### Acceptability and consensus: audio diary analysis, interview analysis and Delphi consensus findings

Two hundred and thirty-eight audio diary sessions were submitted by 14 practitioners, covering 20 cases. Key themes are presented below with illustrative quotes.

Participant SLTs viewed training and supervision as essential for implementation, as did respondents to the SCIP survey.

Training was essential, really important to understand the mapping processes, would be difficult for the integrity of the programme to be maintained without that. (J13^1^ end of therapy reflection)

Access to expert supervision was a key theme related to the optimal implementation of the intervention. Some individuals felt that more time on the initial training would have been helpful, whilst others reported that they needed the experiential learning through starting to use the programme, with regular and timely access to the supervisor. Practitioners wanted a longer training course or access to information online. As practitioners engaged in delivery, they reported becoming more confident in using the manual.

Challenges to intervention delivery were also identified: Some practitioners did not feel equipped to complete the intervention mapping and SCIP-GAS goal setting independently and wanted more time to become familiar with the intervention content. Most participants reported that they had felt very challenged and “stretched” by the very detailed manual, and felt that they were still not sufficiently familiar or confident to use SCIP without reliance on the supervisor.

I completely see the need to have all the activities, but I think it will take a considerable amount of time to become familiar with what I am looking for, and to be able to move around the resource easily. (J16 end of therapy reflection)

Some practitioners reflected on meeting the demands of participating in research over and above therapy delivery:

[it’s been a ]…challenge finding time to deliver and prepare sessions as well as recordings and supervision. (K19)

A strong theme from practitioners was that SCIP is different to current therapy provision, and that protected time to learn the new approach and get to know it were essential. Several practitioners commented that the overall length of intervention was not sufficient. Frequent concerns were reported regarding the time taken to prepare, deliver and write up sessions. Weekly delivery was the maximum that could be provided due to the time required. Participants wanted to adapt SCIP to suit their case or their context, for example, to deliver therapy sessions of less than 1 h duration, extend the duration of intervention, request meetings with school staff for information sharing or to support generalisation.

The SCIP-GAS outcome measure was acceptable to practitioners. SCIP-GAS goals were set by the RSLT, not the practitioners. Practitioners wanted to know the child and the intervention content more thoroughly before setting goals. Some wanted to complete phase 1 therapy before setting GAS goals. They also wanted to carry out the baseline assessments:

Would feel more confident and insightful if I had more involvement in the early stages of assessment and planning….would have felt clearer about delivering intervention if I’d worked jointly with the research team from the start. (K19)

Practitioners wanted to discriminate between GAS scores to report whether a skill was viewed as established or emerging. Participants’ views of the GAS goals had largely changed at time 2, in that having seen progress, they now viewed the goals as appropriate, which caused some to reflect on their own practice.

It’s a shame that it’s only 20 sessions. He has moved up across the board and so many avenues have opened up for further improvement with him, and there is no way he would have had anything like 20 sessions in our usual service. (G05)

Practitioners also reported a range of adaptations and deviations in their implementation of SCIP. These adaptations were predominantly related to time, including shortening sessions and splitting sessions. Most participants reported that they anticipated they would become faster and more confident about personalising resources and activities per child and were positive about continuing to use SCIP with other children. Participants proposed that they would ideally wish to involve the families more directly in the intervention delivery.

Parents valued discussing goals with the RSLT and strongly valued the individualised approach of the intervention:

What I really like about it is having those personalised goals” (A30)

“I love the idea of setting goals rather than just following a format and it being flexible dependent on the child’s needs. (E29)

The SCIP-GAS outcome method was acceptable to parents: all but one completed the form independently:

I really liked the form and the descriptions of changes in skills were really helpful to see and think about. (E27)

Most parents also reported other changes in their child not listed as GAS goals.

He is more motivated and less impulsive. (E27)

He is thinking more about sharing and he said ‘I need to try and think about this’. He’s aware of strategies but he’s not always when he’s in the situation to put them in place. (C16)

Some refinement of the SCIP-GAS rating scale was proposed. Being able to report changes not listed on the SCIP-GAS form as goals is important. One family reported no change on the GAS goals, but listed other important changes for their child as having occurred during the intervention period. Parent priorities were often focused on social “fitting in” and secondary transitions. Parents reflected on the nature of SCIP therapy in bringing about these changes.

It specifically hones in on areas your child needs help with. Rather than previous therapy, which is all a bit generalized. (D18)

Parents preferred delivery of intervention in school time and at school. They perceived the involvement of school in intervention as variable. Additional SCIP work at home was sometimes difficult to incorporate into the family routine.

### Power calculation and Delphi consensus findings

A power calculation for a future trial was carried out using Clsampsi in Stata and SCIP-GAS as the primary endpoint. A 10-point score in SCIP-GAS was clinically meaningful as derived from conservative estimates in this feasibility work; based on a standard deviation of 15, this is a 0.66 effect size. We account for clustering in the SCIP arm with an ICC = 0.01 with 4 SLTs and assume there is no clustering in the treatment as usual (TAU) arm. With 1:1 allocation and 0.05 significance level, a simple two-tailed *t* test with 100 people per group gives 85% power to detect an effect size of 0.5 and 96% power for an effect size of 0.66. In practice, power will be increased by using multiple regression. To allow for 20% attrition in the primary outcome at primary endpoint, we will recruit 250 participants into a trial at baseline split equally across sites.

From the Delphi consensus study, there was 100% agreement that, in a future trial, TAU will be defined by the offer made to children within each service; children should be recruited from NHS and non-NHS services (including schools and independent practices); SLTs will be eligible to participate in a randomised controlled trial if they have protected time to deliver the intervention; training in identifying and managing the needs of children with SCD should be offered to teachers and teaching assistants; training in identifying and managing the needs of children should be provided to parents/carers; the views of children will be sought from those children who are considered capable of engaging with the procedure; the views of parents/carers of children should be sought as part of the trial; and the range and scope of acceptable adaptations to the manualised intervention process and procedures will be clearly defined in the research protocol and controlled in implementation.

There was more than 80% agreement that, in a future trial, SCIP will be delivered in weekly 1:1 sessions by an SLT (with or without assistant); SCIP should be compared to both TAU and/or an alternative controlled programme; engagement by parents and TAs should be defined as an inclusion criterion; an individualised functional measure of the child’s response to therapy should be the primary endpoint; SLTs will be eligible to participate in a randomised controlled trial if they undertake supervision and provide supervision to assistant practitioners; and that SLTs will be eligible to participate in a randomised controlled trial if they undertake training and engage in independent study.

## Discussion

### What we found

SCIP intervention was associated with progress on social communication ratings for all but one of 15 children with SPCD. SLT practitioners valued SCIP therapy and were universal in choosing to continue providing SCIP in their routine practice. They found the intervention complex and needed more preparation and learning time than anticipated to implement it. Parents of children with SPCD valued the intervention highly, and the majority were able to participate in making judgements about outcome independently.

Current provision of speech and language therapy for children with SPCD is highly variable in England, and this will have significant implications for development of a comparison condition in a future trial. There is no current recommended standard of delivery in terms of frequency, method of intervention or mode of delivery. However, SLTs indicated that they were aware of the need for more evidence and showed substantive support for engagement in a clinical trial. The majority of survey respondents were willing to be randomised in a future trial. Time and resources are significant barriers to participation.

Challenges existed in terms of practical aspects of research engagement. SLT services are no longer uniquely commissioned by or provided by the NHS, but are part of a mixed economy of education and health models, with rapid growth of the independent SLT sector also evident. There was a difference in retention and research engagement between NHS and non-NHS services. Non-NHS practitioners had more flexibility to make research-involvement decisions.

SPCD is often included in autism services (with or without diagnosis), making it difficult to isolate as a population. Analysis of national statistics and research literature indicates a potential pool of over 30,000 eligible children with either SPCD or HFA in the 6–11-year age range who could benefit from SCIP. Our power analysis recommends recruitment of 250 (to allow two groups of 100); therefore, sufficient children should be available and eligible to support a larger scale trial. SPCD and HFA-diagnosed children were equally represented in our intervention sample, indicating eligibility for SCIP should be based on need rather than solely on diagnosis.

The SCIP-GAS outcome measure was acceptable to practitioners and parents. Practitioners were able to use descriptions of change to identify progress against targets. Practitioners wanted to know the child and intervention content better before setting goals. Parents valued discussing goals with the Research SLT and strongly valued the individualised approach of the intervention. Parents preferred delivery of intervention in school time and at school. Response rates to parent questionnaires were very high (nearing 100%). Treatment adherence was also very high amongst practitioners.

Analysis of practitioner diaries helped to identify specific areas of support for a future trial.

Practitioners were clear that learning SCIP and implementing it for the first time presented challenges. They indicated that in a future trial, there was a need for additional time to learn a complex intervention based on multiple components, and that additional time would be needed for preparation and recording of intervention sessions. Supervision was essential after initial training; even experienced practitioners were not familiar with therapy methods and planning procedures used in SCIP. However, all practitioners valued the intervention highly and were planning to carry on delivering SCIP in their routine practice in future. SLTs wanted to adapt SCIP to suit their case or their context. Practical suggestions were made for adaptation and revisions to the SCIP manual and resources.

### Limitations

As a single-arm feasibility study, focused on measures and acceptability, there was no comparison group, so it is possible that any appropriate intervention over and above what was currently being offered could be effective. It was not possible to define TAU in this study, since there is considerable variability in practice. TAU will need to be broadly defined and monitored in a future trial to provide a true comparison for SCIP. We underestimated the time practitioners would need to learn and engage with the new intervention, despite their experience, and this resulted in more close supervision being required than anticipated. In future work, practitioners will require more training to support careful and more independent planning as well as time to familiarise themselves with the new complex intervention. A potential source of bias in outcomes is the involvement of parents in the SCIP-GAS procedure. With interventions such as SCIP, it is neither possible nor desirable to have minimum contact between the therapist and the service user, so bias towards reporting of positive effects is possible. In a future trial, it would be essential to distance the reporting of outcomes away from the practitioner and intervention supervisor in the first instance. However, the value of capturing functional outcomes remains. Suggestions for amendments to the GAS procedure are made below.

### Implications for a future trial

Experience of recruitment and feedback from the Delphi study indicated that trial recruitment should be broadly based on social communication need and include all SLT service provision across all sectors. We will identify child participants as having “significant social communication difficulties who will benefit from SCIP intervention” as recommended by the advisory and Delphi panels. This increases the number of eligible participants available to the trial and reflects the sample in the feasibility study. Calculations from recruitment effort in the feasibility study indicate that 250 children can be recruited to the study in two calendar years across the north of England. The inclusion of children with high functioning autism who have pragmatic language impairment will improve the size of the potential sample. There is no counter-indication to this from the outcomes of the feasibility study primary endpoint or from the description of the language needs of children in the current study.

Recruitment would be via NHS SLT services, local education authorities and independent SLT providers. Two gathered cohorts will be recruited in consecutive years. A refined recruitment description will be used based on feasibility feedback. Sufficient time should be included to allow for permissions to be in place across a range of providers for appropriate information, consent and pre-screening to take place. NHS SLT departments tend to be small and have multiple populations to serve other than SPCD. Our experience of recruiting NHS practitioners indicates that direct employment or secondment of SLT practitioners into a trial will provide a more reliable source of basic evidence of effect in a trial whilst English SLT commissioning procedures settle.

The substantive trial would be a two-armed, randomised, controlled, assessor-blinded superiority trial of SCIP versus treatment as usual (TAU) for children, aged 6 to11 years^2^, who have social communication difficulties. This population will include children who have high functioning autism (HFA) and who are able to communicate through spoken language and able to cooperate with intervention. The primary objective would be to compare the effects of SCIP intervention versus TAU on parent completed SCIP-GAS primary endpoint. Since routine practice is highly variable, treatment as usual in a future trial would need to be defined and monitored. In the current study, the Delphi consensus findings recommended that TAU would be defined by the offer made to children with SPCD/HFA within each service. Children in the SCIP condition would be offered a fixed amount of intervention (20 direct sessions) guided by the intervention manual and delivered in weekly 1:1 sessions by SLTs who will undergo a training programme and receive supervision from a RSLT.

The primary endpoint will be the refined SCIP-GAS measure. All participants will be assessed by a research assistant blind to condition. SCIP-GAS goals will be set for all children by research SLTs using parent priorities for intervention. SCIP-GAS change scales would be set but not provided to parents until the end of the intervention period, regardless of randomised group. Time 2 GAS outcomes would be carried out by a researcher independent of the main research team using a scripted procedure and T1-set scale for that individual. GAS goals would be provided at time 1 to the TAU practitioner; any adaptation of TAU aims post GAS sharing would be monitored. A minimum of 2 out of 3 GAS goals scoring at least 2 or more will indicate a clinically significant change from the child’s baseline presentation. If the total score for a child was 3 or below, this would be judged as not clinically significant. These changes should be confirmed by a positive narrative comment from the parent using the following question for each goal: “You gave this goal a score of x. Tell me something you have noticed in your child that makes you think that?”

Parent participation in a future trial is supported by excellent engagement with research questionnaires. In a trial, parent engagement should be supported by training in research participation and SCIP prior to intervention or TAU. Preference for location of intervention was in school, and this was supported by the Delphi panel. In a trial, training and support should be offered to relevant school staff to allow support for research participation. Brief training in SCIP methods for school staff should not exceed similar training provided in TAU. To plan forwards for potential implementation, information should be acquired during a trial regarding the practical arrangements for training and delivering SCIP as part of routine practice. Other recommendations from the Delphi study and from SLT practitioners regarding adaptation of SCIP intervention manual and procedures should be implemented at the start of the trial.

## Conclusions

This feasibility study has provided a crucial step prior to providing definitive data in a follow-up trial. The distinctive nature of the SCIP intervention approach was affirmed by all the participants, with the recognition that this approach may provide an impact for children who have complex needs and who have not benefitted from standard therapy. A trial has a clear potential trajectory into patient benefit with a change in services towards evidence-based practice for children with social communication difficulties called for by service users. A future trial needs to take into account recent changes in speech and language therapy provision and the time pressures associated with research engagement in a small profession. It may be efficient to build a training model to cascade the intervention to SLT practitioners as part of ongoing learning after robust evidence has been developed. There is scope in further work to extend the intervention to other related groups such as children with HFA who use alternative communication devices or children who have secondary pragmatic difficulties associated with learning disabilities.

An innovative primary outcome (SCIP-GAS), based on parent preference, has been refined that meets with requirements of practitioner and service users and has enabled a power calculation for a trial. Initial analysis of associations between narrative outcomes and SCIP-GAS ratings has allowed us to explore the functional impact of the outcome measure. In addition, we now have an appreciation that goal attainment scaling may have wider application for groups with heterogeneous communication needs. Additional service-user secondary outcomes that may be used in a future trial are currently being evaluated and will include a child-perspective interview task and appropriate standardised measures, including parent reported measures.

Qualitative findings underlined the value and acceptability of SCIP intervention to families. We have gained insight into the preferred context and timing of intervention for services. Experience of recruitment, intervention planning, training and supervision has enabled us to make realistic plans for a clinical trial and further implementation into routine practice. This would be the first robust evidence anywhere associated with a complex language pragmatics intervention for this population.

## Data Availability

The datasets used and/or analysed during the current study are available from the corresponding author on reasonable request. Interview and audio-diary data are not available due to the possibility of containing information that could compromise research participant confidentiality.

## Appendix

**Table 4:** SCIP-social communication checklist

**SCIP-social communication checklist (SCIP-SCCheck)**	
**Social communication considerations**	
**(A)** The child has trouble understanding and interpreting the social context and friendship, e.g. social roles and emotions	
**(B)** The child has trouble understanding and/or using nonverbal aspects of communication, e.g. facial expression and intonation	
**(C)** The child has trouble with aspects of conversation, e.g. beginning and ending, taking turns and giving relevant and sufficient information	
**(D)** The child makes bizarre, tangential or inappropriate comments	
**(E)** The child has difficulty using and understanding non-literal language	
**Criteria to progress to screening 2/5 above**

**Table 5:** SCIP-GAS form

SCIP-Goal Attainment Scale (SCIP-GAS) Key Goals (parent version) Sample 9-year-old girl		
	After intervention SCIP-GAS Rating scale Please circle the rating which best describes your child’s current ability	SCIP-GAS comments Please provide comments or examples to support this rating
**Parent stated objective: please note below**	**+ 5** = Increased participation with peers has been noted in more than one additional context **+ 4** = Increased participation with peers has been noted in one additional context **+ 3** = Increased participation in group games with peers at school and/or is withdrawing from group games less often. **+ 2** = Can follow instructions and ask for clarification in role play of playing a game with a group of peers, but has not generalised to peer interactions at school **+ 1** = Has increased awareness of and/or ability to follow instructions and request clarification, but cannot use skill in role play **0** = No change in peer group interaction or in skills associated with peer group interaction **− 1** = is attempting to participate in peer group less often.	
**Example SCIP- GAS goal 1**: Parent: *I’d like her to have someone to invite round after school and that this might lead to a real friendship*		
**SCIP-GAS goal definition:** Please specify the present level and expected achievement		
**Baseline present level** *Does not have a close friend and does not invite anyone home after school.* *Wants to have a friend and enjoys playtime with her younger sister and her friends. Attempts to play with peers in school but mostly unsuccessful and withdraws to be alone.*		
**Expected achievement** *Increased participation in group games with peers at school and/or is withdrawing from group games less often*		

## Abbreviations

ACE: Assessment of Comprehension and Expression
ADOS: Autism Diagnostic Observation Schedule
ASD: Autism spectrum disorder
CCC-2: Children’s Communication Checklist (second edition)
CCG: Clinical Commissioning Group
CELF-4: Clinical Evaluation of Language Fundamentals (4^th^ Edition)
DLD: Developmental Language Disorder
GAS: Goal attainment scale
HFASD: High-functioning autism spectrum disorder
NHS: National Health Service
RPCM: Raven’s Coloured Progressive Matrices
RSLT: Research Speech and Language Therapist
SLCN: Speech, Language and Communication Needs
SCIP: Social Communication Intervention Programme
SCIP-SCCheck: SCIP social communication checklist
SCIP-GAS: SCIP-Goal attainment scale
SLT: Speech and language therapist (or therapy)
SPCD: Social (Pragmatic) Communication Disorder
TAU: Treatment as usual

## Footnotes

1 Participant identification code

2 This age range varies slightly from our previous chosen range (lower limit of 5 years) since we found younger children struggled with some of the SCIP therapy content and its reliance on the written word. The revised age range was agreed with the project steering group.
